# Supplementary figures and images for: Utilization of diabetes management health care services and its association with glycemic control among patients participating in a peer educator-based program in Cambodia
Source: PLoS One. 2020 Jun 25;15(6):e0235037. doi: 10.1371/journal.pone.0235037 (PMC7316287; doi:10.1371/journal.pone.0235037)

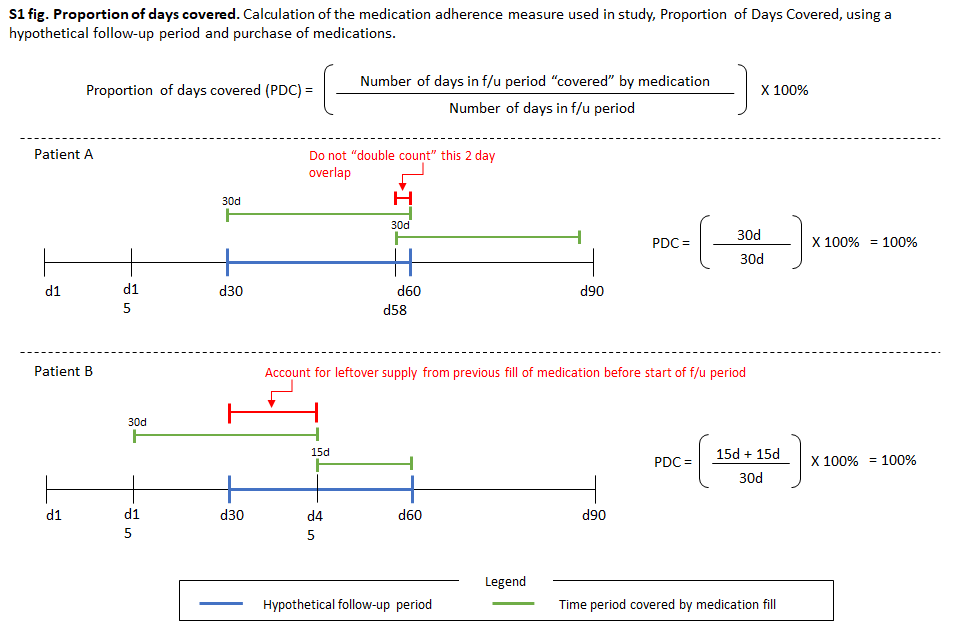

Supplement: S1 Fig — Calculation of the medication adherence measure used in study, Proportion of Days Covered, using a hypothetical follow-up period and purchase of medications. (DOCX) [file pone.0235037.s001.docx]
