# Supplementary material for: Utilization of diabetes management health care services and its association with glycemic control among patients participating in a peer educator-based program in Cambodia
Source: PLoS One. 2020 Jun 25;15(6):e0235037. doi: 10.1371/journal.pone.0235037 (PMC7316287; doi:10.1371/journal.pone.0235037)
Supplement: Attachment — Submitted filename: TD comments (T2D Tx Adherence).docx [file pone.0235037.s003.docx]

**Comments for the study “Treatment adherence among patients with diabetes participating in a peer educator-based disease management program in Cambodia (PONE-D-20-00770)”**

Thank you for giving an opportunity to review this study. Please see below my comments.

**Major comments**

#1 Glycemic control: I have a major concern on the measure of glycemic control in this study. First, I have doubted about the accuracy of post-prandial glucose that should be measured 1–2 h after the beginning of the meal as recommended by ADA, generally peak levels in patients with diabetes. The variation in measure this parameter, specifically not within the recommended time range may occur in the practice of peer educators. Second, I wonder why the authors did not consider HbA1C as an indicator for glycaemic control even lab test for this parameter were also provided to MoPoTsyo members.

#2 Medication adherence: I appreciate the authors attempt to validate data from contracted pharmacies by setting a data quality threshold. However, as the authors mentioned, MoPoTsyo members may purchase their medicines from elsewhere other than contracted pharmacies. The likelihood of underestimated adherence may affect the final model and should not be overlooked.

**Minor comments**

#3 Title: Please consider revise the title, especially the words “treatment adherence”, since it might mislead the readers and does not reflect the context of this study on the four components of MoPoTsyo program.

#4 Abstract: The objective of this study should be clearly defined and consistent with the main texts.

#5 Participant fee: Please clarify if the participant fee for MoPoTsyo program was 5.23 USD per month (Page 6, Line 95), or 5 USD one-time (Page 6, Line 104).

#6 Results: Please present findings of final glycemic control as proportion of participants for each glycemic range.

#7 Discussion: I think it would be more interesting to compare findings from this study with others that explore the effects of peer educator to T2D patients, or to other medical conditions in LMIC.

#8 Discussion: Since adherence to all four components of MoPoTsyo program of nearly half of the participants were generally low, it would be nice if the authors could provide some speculations on this phenomenon.
