# Supplementary material for: Utilization of diabetes management health care services and its association with glycemic control among patients participating in a peer educator-based program in Cambodia
Source: PLoS One. 2020 Jun 25;15(6):e0235037. doi: 10.1371/journal.pone.0235037 (PMC7316287; doi:10.1371/journal.pone.0235037)
Supplement: Attachment — Submitted filename: Response to reviewers.docx [file pone.0235037.s005.docx]

Mayuree Rao, MD

VA Puget Sound Healthcare System

University of Washington

Seattle, WA

Tel: 650-283-2990

Email: [rao.mayuree@gmail.com](mailto:rao.mayuree@gmail.com)

June 2, 2020

Dear Dr. Siyan Yi,

Thank you and your reviewers for your additional comments. Please find below our responses. We are happy to provide any additional clarification if necessary.

Best,

Mayuree Rao

============================================================================

Reviewer #1: I think the authors have appropriately addressed all my previous comments, which made the revised manuscript in line with academic standard and ready for publication. Findings from this study would help strengthening the current evidence to support beneficial impact of peer educators on diabetes care, particularly in deprived settings.

**Response:** none

Reviewer #2: The authors have addressed the comments by making substantial changes to the title, objective, discussion and conclusion.

**Response:** none

Reviewer #3: Thanks for carefully addressing my comments in the previous round, and I am satisfied with your responses. I understand that some limitations cannot be overcome. With this revised version, you have acknowledged those limitations, making the conclusion acceptable. However, I still have three minor comments.

1. On page 5 (line 80), please give a recent update of the coverage.

**Response:** We have added an update to the number of peer educators trained and participants served.

2. On page 6 (line 100), when you said a "small fee", to which standard do you compare (high income living standard?)? In Cambodian rural areas, this amount, to me, may not be small.

**Response:** We have added a sentence to demonstrate that MoPoTsyo’s fees represent a small percentage of monthly household income in Cambodia. If this does not suffice, we can remove the word “small.”

3. On page 13 (line 233), do you have any reference for point '4' saying medication adherence could not be accurately calculated in these instances?

**Response:** We used the specifications established by the National Quality Forum for calculating Proportion of Days Covered, which states that the treatment period must be at least 90 days to calculate this measure. We have added a reference for this.
